# Unusual solar radiation and its impact on the Japanese rice market during the 1830s famine

**DOI:** 10.1038/s41598-026-40316-w

**Published:** 2026-02-18

**Authors:** Mika Ichino, Kooiti Masuda, Takehiko Mikami, Yasuo Takatsuki

**Affiliations:** 1 Center for Open Data in the Humanities, Joint Support Center for Data Science Research, Research Organization of Information and Systems, 3-10, Midori-cho, Tachikawa-shi, Tokyo, 190-0014 Japan; 2 Faculty of Geo-Environmental Science, Rissho University, 1700 Magechi, Kumagaya-shi, Saitama, 360-0194 Japan; 3 Tokyo Metropolitan University, 1-1 Minami-Osawa, Hachioji-shi, Tokyo, 192- 0397 Japan; 4 Research Institute for Economics and Business Administration, Kobe University, 2-1 Rokkodai, Nada, Kobe, 657-8501 Japan

**Keywords:** Climatic impact, Economic fluctuation, Tenpō famine, Solar radiation, Japan, Rice, Climate-change impacts, Environmental economics

## Abstract

We investigated the interplay between climatic anomalies and economic fluctuations in early modern Japan (1603 − 1867), focusing on the Tenpō Famine of the 1830s. Specifically, we reconstructed solar radiation from 1821 to 1850 using descriptions from 18 historical diaries. This represents a novel approach to analyzing climatic impacts on agriculture and the economy during this period. The results suggest that reduced solar radiation, indicative of poor weather conditions, was followed by a substantial increase in rice prices, which rose to three to four times the normal level (from 50-70 to around 200). This rise occurred particularly during the summer of 1836, when solar radiation in July and August was approximately 10% below normal, and cold conditions persisted for three months. Applying principal component analysis to the reconstructed solar radiation data revealed spatiotemporal patterns that elucidated the link between climatic anomalies and their impacts on market prices during the Tenpō Famine. This indicates the sensitivity of market prices and economic stability to climate fluctuations. Using monthly rather than annual data, this study offers further evidence for the connections between climate, agriculture, and economic fluctuations. Our findings provide historical perspectives that could contribute to broader considerations in climate adaptation and policymaking. Additionally, this study suggests further research directions and encourages continued exploration of the relationship among climate change, agriculture, and economic fluctuations, inspiring future research in this field.

## Introduction

How do societies respond to varying climates? This question not only arouses academic curiosity, but also leads us to understand how our society can adapt to ongoing climate change. We approached this issue by focusing on early modern Japan (1603–1867), which experienced severe famine due to poor harvests caused by an abnormal climate. Indeed, early modern Japanese society was vulnerable to abnormal climates; hence, investigating such occurrences can provide insights into vulnerable areas in the modern era.

We will focus on the 1830s, when the Tenpō Famine, one of the greatest famines in Japanese history, occurred. The Tenpō Famine provides an ideal case study because it affected the entire country while exhibiting distinct regional variations, and because historical data, such as diaries and rice price records, are relatively abundant and accessible. During the Tenpō Famine, many people were starved to death, particularly in the northeastern region^1^. However, the degree of damage varies even in the northeast region^2^. Therefore, it is necessary to closely examine the weather in each region.

Historical climatology has developed significantly through proxy data, such as tree rings, lake sediment cores, and historical records, providing insights into past climatic conditions, particularly for the pre-19th century and early modern periods. Brázdil et al.^3^summarized European climate reconstructions using these proxies, while Neukom et al.^4^analyzed regional climate patterns and the factors influencing them, including external influences. Brönnimann et al.^5^highlighted the 1835 Cosigüina volcanic eruption, which released a large amount of sulfur dioxide into the stratosphere, as a potential contributor to the abnormal climate after the eruption. These studies demonstrate the value of historical data in understanding global and regional climate variations. Hirano and Mikami^6^reconstructed the winter climate data for Japan, whereas Mikami^7^focused on summer climate reconstruction. These studies have utilized historical weather records to clarify the characteristics of climate change.

However, traditional proxy data, such as tree rings and lake sediments, often lack the seasonal and regional resolutions necessary for a detailed analysis of historical climate variations. Furthermore, previous reconstructions based on historical diaries were limited in their temporal scope. They lacked continuous year-round data or coverage across multiple seasons and regions, leaving gaps in our understanding of climate variation at finer temporal and spatial resolutions.

Ichino et al.^8^reconstructed monthly solar radiation data for 1821–1850 to address these limitations based on historical weather records. Their study produced year-round data from 11 locations across Japan, which enabled the identification of regional solar radiation patterns. This dataset provides a critical foundation for examining the relationships between climate variation, agricultural production, and economic indicators such as rice prices. Nishimori and Yokozawa^9^demonstrated that rice yields in western Japan are highly sensitive to solar radiation, whereas temperature plays a more significant role in the Tohoku region, in the northern part of the Japanese mainland. These findings underscore the importance of Ichino et al.’s data in understanding the impacts of climate variation during events, such as the Tenpō Famine.

There has also been a growing interest in the relationship between climate and the economy. Brunt^10^demonstrates that weather had a significant influence on annual English wheat yields (1690–1871), distorting historical productivity estimates. Using U.S. historical records (1860–2000), Bleakley and Hong^11^show that hotter and wetter conditions reduced farm output in the late nineteenth century, but these adverse effects have nearly disappeared in recent decades, reflecting effective adaptation through technological and environmental improvements. Rönnbäck^12^reconstructs price series for corn, yams, and palm oil on the pre-colonial Gold Coast (1699–1760), showing that markets responded sensitively to climate variation, crop resilience, and external shocks such as wars.

These studies provide valuable insights into the connection between climate change and agricultural production, but their annual resolution makes it difficult to pinpoint when and how people recognized climate shifts. In contrast, our paper uses both monthly solar radiation data and monthly rice price data to examine more closely how people perceived climate variability and how that perception influenced their economic activities.

Although rice prices, which reflect the early modern Japanese economy, were traditionally recorded only once a year in the 12th month of the Japanese lunar calendar, we can now access daily and monthly data, enabling more detailed observations. This development allows us to go beyond previous research on the link between climate and society in the 1830s. Most studies on the Tenpō Famine are qualitative, and the only one that utilizes paleoclimatic and quantitative economic data is Hamano^13^. At a one-year resolution, he analyzed how climate affected food availability and demographic changes in Japan during the 1830s. He showed that cool summers in 1833, 1836, and 1838 led to food shortages in the following winters and to decreased birth rates in 1834 and 1837. He proposed a causal relationship among these three factors, which should be revised in at least two ways.

To determine the climatic conditions, Hamano^13^used weather information compiled from diaries in the Historical Weather Database^14^and considered the number of rainy days in July to indicate summer temperatures, as demonstrated by Mikami^15^for Tokyo. Hamano^13^reported that there were only a few rainy days in July 1836 in the Tohoku region. However, circumstantial evidence suggests that the temperature was lower during this period; this indicates that the number of rainy days cannot be converted to temperatures in the Tohoku region of Japan.

Second, to reflect food supply and demand, Hamano applied rice prices in Osaka for the 12th month of the Japanese lunisolar calendar year, as compiled by Iwahashi^16^. However, as explained later, there is a problem with using the rice price in the 12th month of the year to represent that year’s price. From spring, when rice planting begins, to autumn, when the harvest is gathered, people trade rice considering the weather. The market’s reaction to weather and the forecast for the rice harvest cannot be reconstructed from the prices of the 12th month alone. A pioneering study by Hamano^13^identified various unresolved issues; however, follow-up studies have not been performed.

Understanding climate change and its devastating impacts on societies in the past requires social and economic information regarding such periods; this involves reconstructing spatial patterns of climate variation at a higher temporal resolution than that provided by annual data. More detailed climatic and economic data can guide discussions on climate variation and its effects on historical societies. Therefore, this study sought to reconstruct the monthly mean solar radiation data from 1821 to 1850 based on weather descriptions recorded in 18 historical diaries. It also discusses the abnormal seasonal climate and its economic effects, focusing on the Tenpō Famine in Japan in the 1830s.

This study consists of three main steps. First, we reconstruct monthly solar radiation from historical weather descriptions recorded in 18 diaries across Japan. Second, we analyze spatiotemporal structure using principal component analysis (PCA). Finally, we examine how these climatic variations correspond to fluctuations in rice prices during the 1830s famine period.

## Methods

### Reconstruction of solar radiation patterns

#### Data

**Fig. 1:**
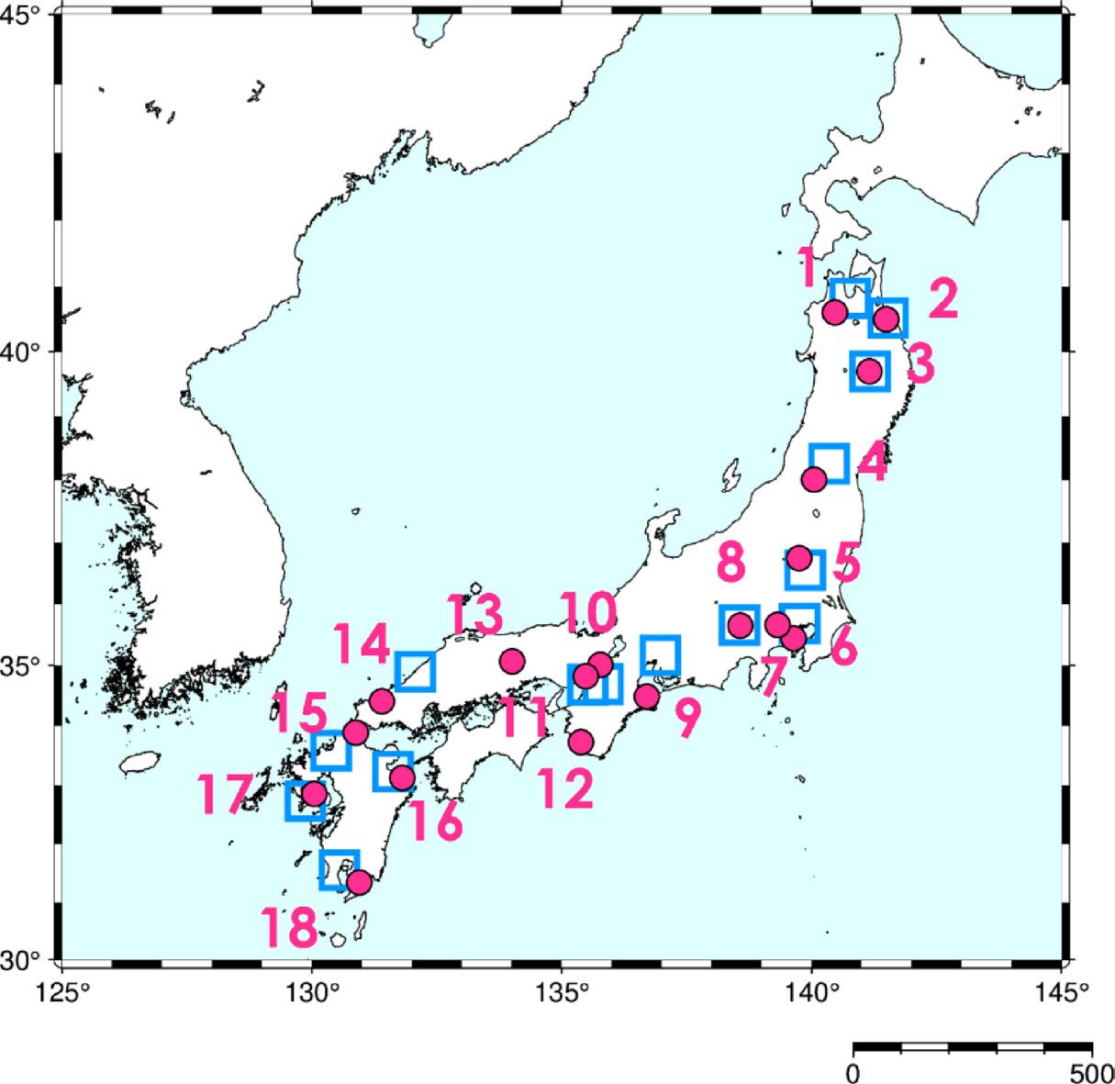
Location points of the historical diaries and Japan Meteorological Agency (JMA) observatories were utilized in this study. The red circles represent historical weather recording locations listed in Table 1. 1, Hirosaki; 2, Hachinohe; 3, Morioka; 4, Kawanishi; 5, Nikko; 6, Yokohama; 7, Hachioji; 8, Kofu; 9, Ise; 10, Kyoto; 11, Ikeda; 12, Tanabe; 13, Tsuyama; 14, Hagi; 15, Kitakyushu; 16, Usuki; 17, Isahaya; 18, Koyama. The blue square frames indicate the Japan Meteorological Agency observatories used for estimations. 1: Aomori, 2: Hachinohe, 3: Morioka, 4: Yamagata, 5: Utsunomiya, 6: Tokyo, 7: Tokyo, 8: Kofu, 9: Nagoya, 10: Nara, 11: Osaka, 12: Osaka, 13: Nara, 14: Hamada, 15: Fukuoka, 16: Oita, 17: Nagasaki, and 18: Kagoshima. This map was created by the authors using PyGMT (version 0.7.0; https://www.pygmt.org) and Matplotlib (version 3.6.2; https://matplotlib.org).

Daily weather descriptions from 18 historical diaries from 1821 to 1850, over 30 years, were collected from the Historical Weather Database (HWDB) developed by Yoshimura^17^. In the HWDB, dates were translated from the Japanese lunisolar calendar to the Gregorian calendar. Table 1; Fig. 1 show the locations where the data were recorded. Although the dataset includes only 18 sites, which would be seen as sparse in today’s meteorological observation network, it is one of the most extensive collections of historical weather observations for the early modern period. This makes it a valuable proxy for examining the large-scale climate patterns at that time.

**Table 1:** Locations of historical diaries and Japan meteorological agency (JMA) observatories utilized in this study.

Historical Diary No.	Historical Diary Location	Latitude (°*N*)	Longitude (°E)	Recording Period	JMA Observatory Location	JMA Observation End^a^
1	Hirosaki	40.61	140.47	1661–1867	Aomori	
2	Hachinohe	40.50	141.49	1792–1867	Hachinohe	September 2007
3	Morioka	39.70	141.16	1661–1840	Morioka	
4	Kawanishi	38.00	140.05	1830–1889	Yamagata	
5	Nikko	36.75	139.75	1685–1871	Utsunomiya	
6	Yokohama	35.44	139.64	1806–1889	Tokyo	
7	Hachioji	35.66	139.32	1720–1885	Tokyo	
8	Kofu	35.65	138.57	1747–1872	Kofu	
9	Ise	34.48	136.70	1683–1889	Nagoya	
10	Kyoto	35.01	135.77	1796–1866	Nara	
11	Ikeda	34.82	135.48	1714–1892	Osaka	
12	Tanabe	33.72	135.38	1814–1869	Osaka	
13	Tsuyama	35.01	135.77	1702–1868	Nara	
14	Hagi	34.41	131.40	1739–1867	Hamada	September 2007
15	Kitakyushu	35.07	134.01	1811–1857	Fukuoka	
16	Usuki	33.88	130.88	1674–1868	Oita	
17	Isahaya	32.85	130.05	1700–1868	Nagasaki	
18	Koyama	31.34	130.95	1825–1871	Kagoshima	

^a^ JMA Observations available up to the month and year noted.

The historical diaries provide weather descriptions; the JMA observatories serve as sources of solar radiation and *Tenki-gaikyō* (daily weather conditions) data during the daytime.

The solar radiation and daytime weather conditions recorded at these locations by the Japan Meteorological Agency (JMA) from 1981 to 2010 were used to calculate the conversion parameters. The daily observed weather conditions from the JMA contained “*Tenki-gaikyō*” (a weather summary) twice daily, once during the day and once at night. These data were generated by local observatories following the method described in the Guidelines for Surface Weather Observation Statistics^18^. Daytime *Tenki-gaikyō* was applied in the current study and was assumed to be the most similar to the historical daily weather records. These data were generated by summarizing “*Tenki*,” a combination of cloudiness- and precipitation-related parameters recorded every three hours from 06:00 to 18:00.

Daily total solar radiation (***S***) data from JMA’s daily surface weather observations were used. The solar radiation reported in the Annual Report of the JMA from 1981 to 2010 was obtained from https://www.data.jma.go.jp/risk/obsdl/ and used to determine the conversion parameters. Table 1; Fig. 1 show the JMA observatories from which these data were available for estimating historical solar radiation.

#### Reconstruction of solar radiation patterns

Although historical weather records have been used to reconstruct data related to historical climatic variations in various parts of Japan during specific seasons (e.g., summer or winter)^6,7^, few studies have estimated monthly or daily climatic parameters, regardless of season or location, using proxy data, such as historical weather records, tree rings, and lake sediment cores. Our group presented a schematic for estimating solar radiation based on weather descriptions. These weather descriptions include reports of clouds or rainfall between the sun and an observer standing on the ground, which can indicate the degree of reduction in the incident solar radiation caused by clouds and other atmospheric aerosols. Solar radiation is closely related to weather conditions as described in the weather records. Hence, weather descriptions reflect the amount of solar radiation on the Earth’s surface. Accordingly, solar radiation, particularly the total downward solar radiation at the surface, was selected as the target variable for estimation.

This study estimated historical solar radiation using written and verbal expressions of weather conditions from historical documents, namely, diaries and observational logbooks. Weather descriptions were categorized into three levels and converted into solar radiation using conversion parameters determined from modern observations (Table 2). As previously explained, historical diaries and JMA’s modern weather descriptions, including solar radiation recorded using instruments, are necessary for reconstructing solar radiation patterns.

**Table 2:** Specifications of weather levels and the classification of weather descriptions.

Type	Category of weather description	HD^a^	TG^b^
**Weather level k**	Fine	1	1
	Fine, partly cloudy	2	
	Half fine, half cloudy		
	Cloudy, partly fine		
	Cloudy/Half fine, half rainy		2
	Cloudy, partly rainy		3
	Half cloudy, half rainy		
	Rainy, partly cloudy		
	Rainy/snowy	3	

^a^ Type HD: For weather descriptions in historical documents.

^b^ Type TG: Weather descriptions from the Japan Meteorological Agency, *Tenki-gaikyō*.

The method used in this study was derived from the work of Ichino et al.^19^, who developed an estimation method that converted *Tenki-gaikyō* into solar radiation values. Daily total solar radiation (*S*) was obtained from the daily JMA surface weather observations. Normalized *S* is referred to as *q* and is defined by Eq. (1):

1 $$q\,=\,S/{S_{{\mathrm{TOA}}}}$$

where *S*_TOA_ is the daily insolation received at a horizontal surface at the top of the atmosphere and is computed using the equations described by Kondo and Xu^20^. Although it varied throughout the historical period, we consistently applied the recent value of 1.365 × 10^3^ W/m^2^ as the total solar irradiance (TSI) to calculate the *S*_TOA_ throughout this study.

The average ratio of *q* for each month to weather level *k* is referred to as *q*_*mean*_*(k)*, which was calculated at each location where *Tenki-gaikyō* and solar radiation were used (Table 1; Fig. 1). We speculate that the *q*_*mean*_*(k)* values for the 19th century and 1981–2010 were identical.

*Tenki-gaikyō* was classified into three *Tenki-gaikyō* (TG) levels. The nine categories of *Tenki-gaikyō*^19^and the two types of classifications are listed in Table 2. Categories describing weather conditions were established according to the method described in^19^, and named according to the common descriptions found in *Tenki-gaikyō*, ranging from the most favorable (fine) to the least favorable (rainy or snowy). These categories were designed to organize a wide range of weather descriptions systematically. For instance, “rainy or snowy” weather conditions include rain, snow, graupel, hail, and sleet.

**Fig. 2:**
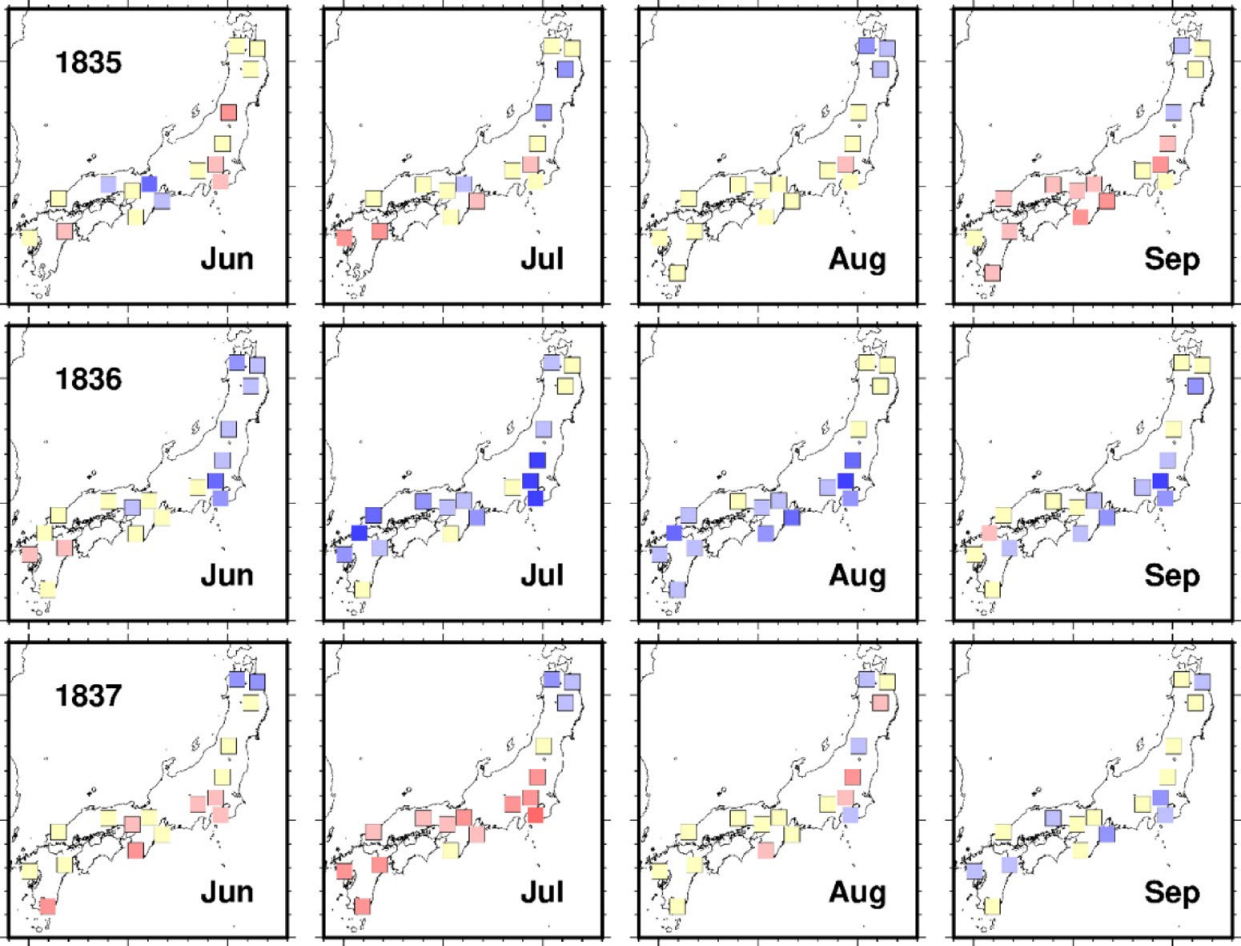
Distribution maps of reconstructed monthly mean solar radiation at 18 locations across Japan. These values represent the ratios of estimates from January to December in 1835, 1836, and 1837 to the average over 30 years, from 1821 to 1850. These maps were created by the authors using PyGMT (version 0.7.0; https://www.pygmt.org) and Matplotlib (version 3.6.2; https://matplotlib.org).

Solar radiation and weather conditions are strongly correlated. Therefore, we initially explored the quantitative relationship between *q* and *Tenki-gaikyō*. The distribution of *q* for each weather level in Tokyo (August, 1981–2010) has already been documented in Ichino et al. [21, Fig. 2], demonstrating a clear decrease of *q* from fine to rainy or snowy conditions. Consequently, the weather levels deduced from weather descriptions were converted into solar radiation using Eq. (2):

2 $${S_{ej}}={\text{ }}{q_{mean}}\left( {{k_j}} \right)*{\text{ }}{S_{TOAj}}$$

where *S*_*ej*_ is the estimated solar radiation, *j* is the day, and *k*_*j*_ is the weather condition indicated by “weather level.” *S*_TOA *j*_ is the daily solar radiation that reaches the top of the atmosphere on day *j*, and *q*_*mean*_*(k*_*j*_*)* is a conversion parameter determined by weather level *k*_*j*_ and calculated using weather observations recorded at the JMA from 1981 to 2010.

​In this study, we used the monthly mean *S*_*ej*_ as a climate variable because the errors in *S*_*ej*_ relative to the observed *S* were not small enough for *S*_*ej*_ to be used as daily data. However, the variation in *S*_*ej*_ was similar to that observed for *S*, and the verification in^21^demonstrated that the daily root-mean-square error (RMSE) was approximately 30 W m⁻². When averaged monthly, the estimates of *S*_*ej*_ achieved accuracy comparable to those derived from sunshine duration (RMSE ≈ 8 W m⁻² vs. 7–8 W m⁻²; *r* = 0.977, mean absolute error = 6.25 W m⁻², relative RMSE ≈ 5.9%). In addition^19^, validated the estimates for Tokyo and several other locations in Japan (Nagasaki, Saga, Takada, Sendai, Yamagta, Aomori, and Hachinohe), obtaining RMS errors of approximately 6–10 W m⁻² (relative error 4–9%) for Tokyo, and 3–7 W m⁻² (relative error 3–6%) for the other locations. This confirms that using monthly mean values effectively reduces the uncertainty inherent in daily weather-level classifications.​

Historical weather descriptions were categorized into appropriate weather levels, *k* to estimate historical solar radiation and *Tenki-gaikyō*. Based on^21^, we adopted the three-level HD classification shown in Table 2, which was developed by comparing weather descriptions from historical diaries with *Tenki-gaikyō* and examining their characteristics.

As shown in Table 2, fine was designated as weather level *k* = 1, rainy as *k* = 3, and snowy, graupel, hail, and sleet were considered equivalent to rainy (*k* = 3). As mentioned in the typical weather expressions for the categories, *k* = 2 includes other weather descriptions, unlike in *Tenki-gaikyō*. For example, temporary clouds in historical diaries were categorized as *k* = 2, whereas the same weather descriptions in *Tenki-gaikyō* were classified as *k* = 1. In historical diaries, a rainy day with intermittent rain was also categorized as *k* = 2. However, in *Tenki-gaikyō*, it was categorized as *k* = 3.

#### Parameter determination with modern observations

To calculate *q*_*mean*_*(k)*, observed weather conditions and solar radiation are required. However, instrumental observations of solar radiation and weather conditions were rarely available at the same location where the weather was recorded in the historical diaries.

Ichino and Mikami^22^examined the spatial applicability of *q*_*mean*_*(k)* for estimating monthly mean solar radiation and found that using stations within the same Japan Weather Association (JWA) climatic division^23^yields smaller estimation errors than using geographically closer stations.

These results allowed the JMA stations to be selected based on the JWA climatic divisions of solar radiation. The JWA climatic divisions are defined by similarities in seasonal patterns of solar radiation among regions. The observational data from the JMA locations listed in Table 1; Fig. 1 were used to calculate the parameters.

#### Analysis of the Spatiotemporal structure of solar radiation

To determine the spatial and temporal scales of the variation in solar radiation, we performed Principal Component Analysis (PCA). The procedure ‘prcomp’ of R 4.3.0 was used with its default options. Its input consisted of monthly values of the normalized solar radiation *q* for one month (June, July, August, or September) from 1821 to 1850 at 18 locations which are shown in Table 1. The output consists of 18 components, each of which is a pair of an eigenvector and a sequence of its scores representing the variation’s spatial and temporal structures.

Only such sets of observations that have valid values at all locations can contribute to constructing the eigenvectors. However, the *q* value was missing in some locations in some months because the number of days with valid records was less than 2/3 of the total days in the month. We tentatively replaced missing values with zero anomalies; that is, we assumed that *q* was the same as the average of the 30 years. This assumption is likely to underestimate the actual variation. However, it is unlikely that false signals will be generated.

### Rice price

The method used in this study to estimate solar radiation during past periods can be applied throughout Japan regardless of the specific season or region. It can also be used to estimate the climate during periods of less than one year and seasonal changes. Such an approach will supplement the discussion on human society and climatic factors, as it provides data better suited to the temporal resolution of social change than other proxy data that are limited to specific seasons (e.g., summer or winter).

Furthermore, our method enables the estimation of solar radiation from daily weather descriptions at various temporal resolutions, allowing for averaging over specific periods, such as months, the growing season of a crop, or an integrated value rather than a simple average value over a set timeframe, as well as the estimation of annual totals. The flexibility of our method enables comparisons across diverse indicators, such as production, yield, price, and other economic factors.

To confirm this, we compared the reconstructed solar radiation with rice production. Data on agricultural yields are required to rigorously understand the social impacts of climate change. However, although historical records of accurate yields have been preserved in minimal areas, no historical statistics are available to determine the yields for the entire country. Therefore, previous studies have used prices as a proxy for output, especially rice prices, for which the most continuous data are available; this study follows suit. Contemporary records, introduced in the later section, clearly show that poor rice harvests led to rising rice prices. Therefore, it is reasonable to use rice prices as a proxy indicator for rice production in this context.

Rice was not only the staple food of the time but also a symbolic good of early modern Japan. Rulers collected rice as a tax and sold it to the market to generate financial revenue. Therefore, continuous time-series data on rice, among other grains, have been preserved in historical documents.

However, caution must be exercised when using rice prices as a proxy indicator. First, we must ask whether trade with other countries caused fluctuations in rice prices. However, this question does not apply to the present study. From 1639 until its collapse in 1867, the Tokugawa Shogunate imposed a strictly controlled trade regime prohibiting rice import and export. After 1639, rice was produced throughout Japan and consumed domestically without being exported or imported^24^. The national population did not change considerably during the observation period^25^. Additionally, according to Arizono^26^, who studied the dietary habits of peasants in the early modern period, although detailed information for the 17th century isn’t available, it is known that rice consistently remained the main food for peasants from at least the mid-18th century onward. These facts suggest that consumer demand remained unchanged during the period. Thus, it appears that the primary supply factors (yield) affected the price of rice, and the climate affected its supply.

Second, we should consider whether other events, such as riots, typhoons, or government price controls, have a significant impact on rice prices. Regarding riots in 1837 in Osaka, which had the most extensive rice market at the time, one official incited a large-scale riot to denounce the inadequacy of the shogunate’s rice price reduction policy and the injustice of other officials. This riot upset rice prices and should be carefully considered when observing later fluctuations in rice prices; however, we conclude that its impact was limited. Rice prices rose immediately after the riots, but the effect was short-lived, partly because the riots were suppressed within one day. Instead, we believe the upward pressure on rice prices was exerted by the lack of solar radiation after the riots.

In the 1830s, the shogunate instituted various measures to reduce rice prices. For example, stockpiled rice was released, and orders were given to local lords to bring more rice to Osaka and Edo (Tokyo). However, since these measures were ineffective and in some ways triggered the riots mentioned earlier, it is reasonable to conclude that they did not significantly impact the price of rice.

Therefore, using rice prices as a proxy indicator of rice output is acceptable. Prior studies, such as Hamano^13^, use rice prices as a proxy indicator to understand rice production. He applied the rice price in Osaka in the last month of the Japanese lunisolar calendar year, provided by Iwahashi^16^, rather than the average yearly price to reflect food supply and demand. However, there are problems with this approach.

**Fig. 3:**
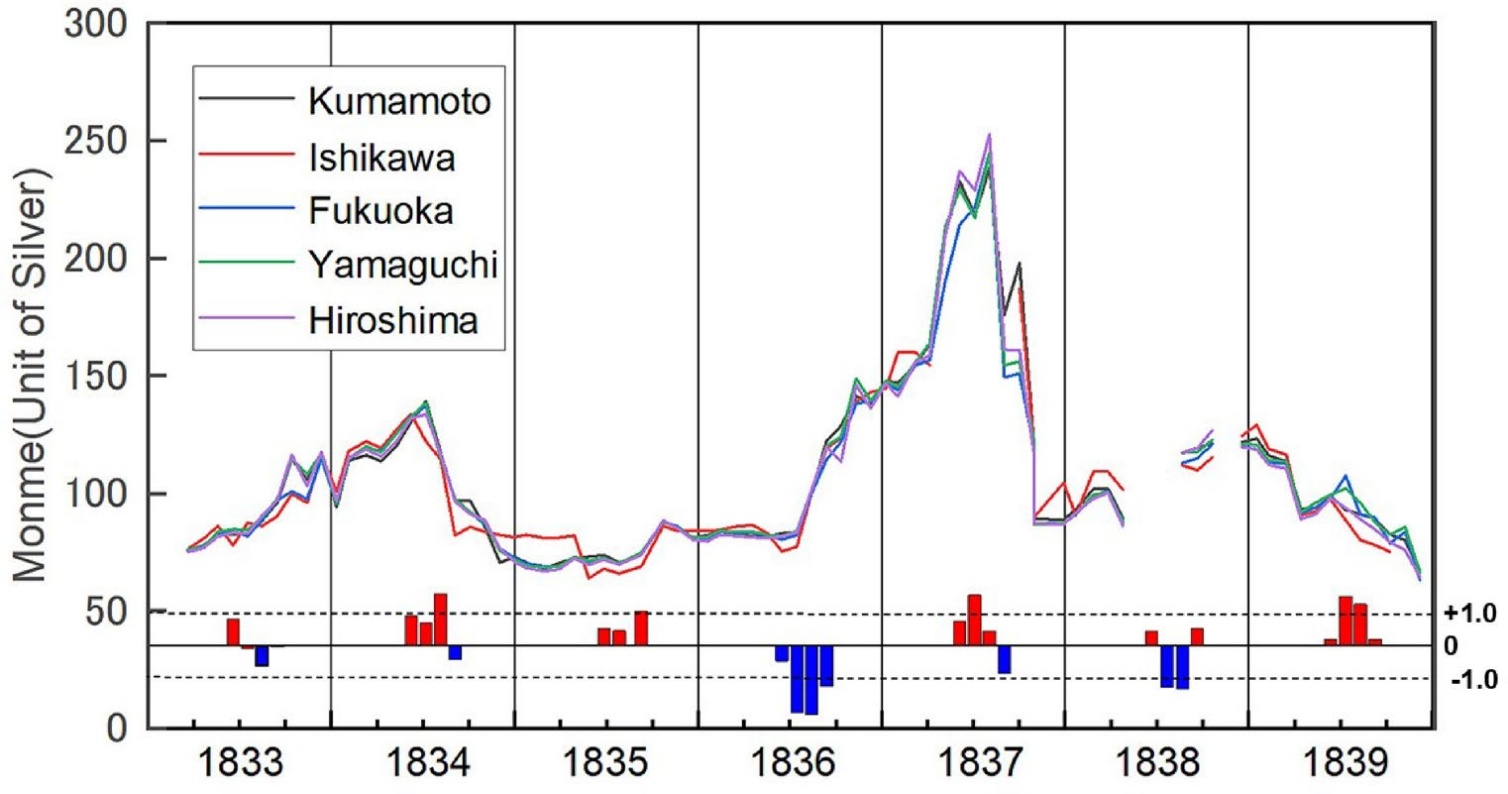
Monthly rice price from 1833 to 1839 in Osaka. (Source) “Sho Sōba no Hikae” (a record of market activities) stored in the archives of the Mitsui Group in Tokyo^27^. Each marker on the graph represents a monthly price converted from a lunisolar calendar to a solar calendar. Since 1835 and 1838 include leap months, the conversion to the solar calendar results in 13 months. Monme was the standard unit of value used in Osaka at the time. The PC1 score displays the values for each year, spanning the four months from June to September. Lower values of PC scores indicate less solar radiation.

Rice is harvested in the 9th or 10th month (around October or November in the solar calendar) of each year; hence, the rice price in the 12th month is generally representative of the rice price for that year. However, this was not the case in years with poor harvests. As shown in Fig. 3, a poor crop in year *t* can lead to higher prices in the summer of year *t* and in the spring and summer of the following year (*t + *1). Prices rise in the summer, before the rice harvest (in autumn), because rice merchants have a nationwide information network about rice crops. During the summer, they trade rice based on the expected annual harvests of the year^24,28^. Suppose that rice production recovers in year *t + 1* and prices in the 12th month in year *t + 1* would be lower, and that by the 12th month in year *t + 1*, prices have lowered. In that case, year *t + 1* would be understood to have been a year of low rice prices, even though the first half of year *t + 1* suffered from high rice prices. Thus, in a bad harvest year or year after the bad harvest year, the price of rice in the 12th month should not be considered representative of that year’s price.

**Fig. 4:**
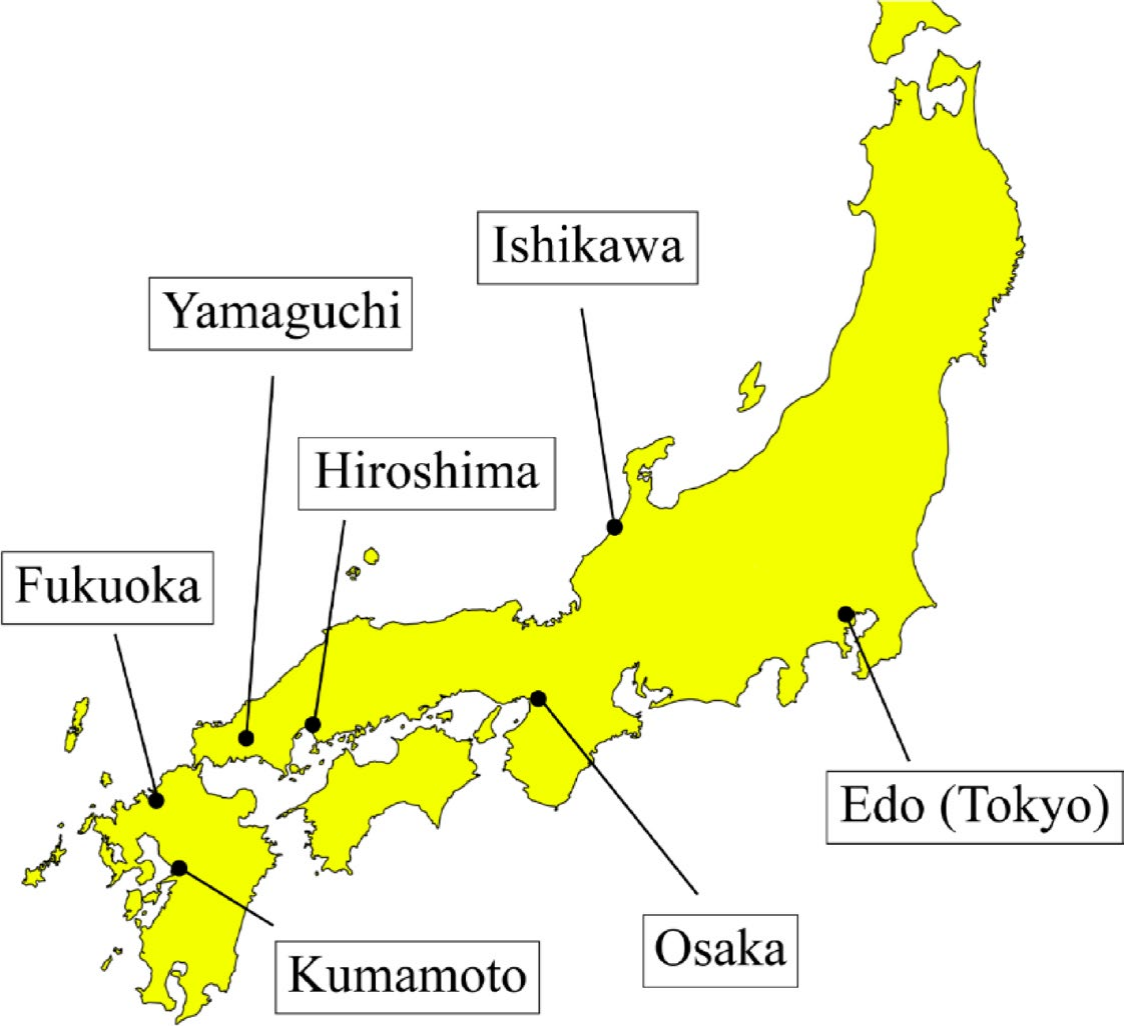
Historical map of early modern Japan. Rice produced in the areas shown here was transported to Osaka by sea and traded under names such as Kumamoto rice and Ishikawa rice. The rice prices we are referring to here are not those for local areas such as Kumamoto and Ishikawa, but rather those set in the central rice market in Osaka. The base outline map was derived from SimpleMaps (© SimpleMaps, CC BY 4.0, https://simplemaps.com), and the figure was created and edited by the authors using Adobe Illustrator (Adobe Inc., version 29.8.4, https://www.adobe.com/).

To update the pioneering work of Hamano^13^, we refer to monthly rice price data from the “Sho Sōba no Hikae” (a record of market activities) stored in the archives of the Mitsui Group in Tokyo^27^. The database contains monthly reports on the prices of essential products. Among these records, we evaluated documents from 1833 to 1839 that recorded the monthly price of rice in Osaka. In principle, monthly price refers to the price at the beginning of each month. We converted these from a lunisolar calendar to a solar calendar. Here, we refer to the price of rice produced in Kumamoto, Ishikawa, Fukuoka, Yamaguchi, and Hiroshima (Fig. 4). Approximately 30 brands of rice, differentiated by production area, are traded in the Osaka rice market, of which five are leading brands^24^.

The brands’ prices referred to here are not those in a specific region, such as Kumamoto, but the prices set in the Osaka rice market. As rice produced in Kumamoto is sent to Osaka and traded in the central market, the Osaka rice market, it was also considered at the time to reflect the trends in supply and demand nationwide^28^, just as Toyota’s stock price is deemed to be a proxy index that indicates the economic situation of the whole of Japan rather than the financial situation of Toyota City, where Toyota’s headquarters is located.

There is a reason why the rice brands we refer to are biased towards Western Japan. Rice produced in Eastern Japan was transported to Edo by sea and traded there; however, unlike in Osaka, wholesale traders traded rice on a one-to-one basis, and prices were not widely publicized. Therefore, obtaining time-series data on rice prices in Edo is almost impossible. However, this bias did not hinder the analysis. It has been pointed out that the price of rice in Edo was highly dependent on the price of rice in Osaka^28^. It is also known that the government (the shogunate) used the Osaka rice price as a benchmark when implementing policies to adjust rice prices^24^. Therefore, the prices of the five brands referred to here represent rice supply and demand.

## Results

### Reconstructed solar radiation patterns

We reconstructed the monthly mean solar radiation from 1821 to 1850, based on weather descriptions from 18 historical diaries. Here, we assume that the *q*_*mean*_*(k)*, which is the average of *q* for each weather level, is the same for the diaries during the historical period as for the current weather conditions of the JMA. Our analysis focused on 1836, marked by the most severe famine in the Tenpō era. Although frequent cool summers and crop failures have been suggested as the causes of famines, the mechanisms that trigger these events remain unclear.

Figure 2 illustrates the reconstructed solar radiation anomalies from 1835 to 1837 as monthly deviations from the 30-year mean (1821–1850). The maps indicate a significant reduction in solar radiation in the summer of 1836, especially in central Japan, which is aligned with the severe economic conditions of the Tenpō Famine. Although daily estimates were initially generated, monthly averages were used to minimize estimation errors. Only the months with at least 20 recorded diary entries were included to ensure data reliability. This methodology reduces uncertainties arising from biases in diary weather descriptions or disparities between the diary records and *Tenki-gaikyō*.

During the summer of 1836, solar radiation in the east-west zone of Japan, including Kanto, Kinki, and northern Kyushu (locations 5–15), decreased significantly, approximately 10% below the historical average for July and August. In contrast, the solar radiation levels in Tohoku (locations 1–4) to the north and southern Kyushu (location 18) to the south remained relatively stable, highlighting the distinct regional variations in solar radiation patterns. The reconstructed solar radiation levels from May to September 1836 were consistently lower across Central Japan. In contrast, the solar radiation levels in spring (February–April) and autumn (September–November) were within the average range.

### Spatio-temporal structure of solar radiation as revealed by principal component analysis

**Fig. 5:**
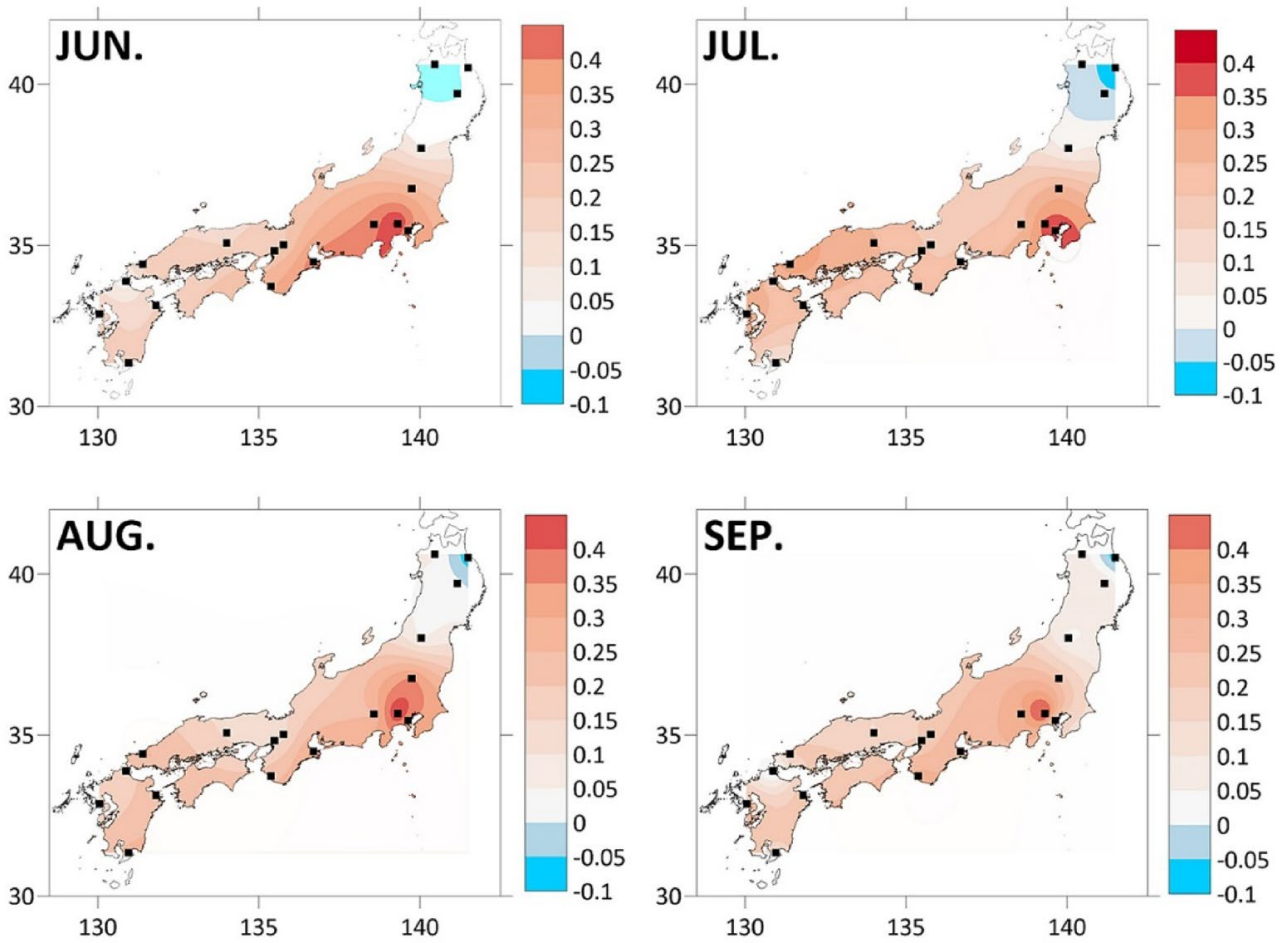
Eigenvector of the PC1 of monthly solar radiation in 1821–1850. Black dots indicate the locations of diaries in the map. (a) June. (b) July. (c) August. (d) September. Note that the sign of the eigenvector for September was reversed. The contour map was created by the authors using Surfer (Golden Software, version 28; https://www.goldensoftware.com).

The PCA of ***q*** was performed as described in Sect. 2.2. The proportions of variance in the first principal component (PC1) were 39, 42, 46, and 31%, respectively, for June, July, August, and September. The eigenvectors of PC1 for each month are shown in Fig. 5 (a-d). In all four months, the signs were almost the same everywhere. Opposite signs occur in the Tohoku region (north of 38 °N), but the magnitude is not large there. For convenience of comparison, the sign of the eigenvectors is so taken that the values are positive in most of the locations. When the score of PC1 is negative, solar radiation is weaker than normal in the zone around 32–37 °N, such as from Kyushu to Kanto.

**Fig. 6:**
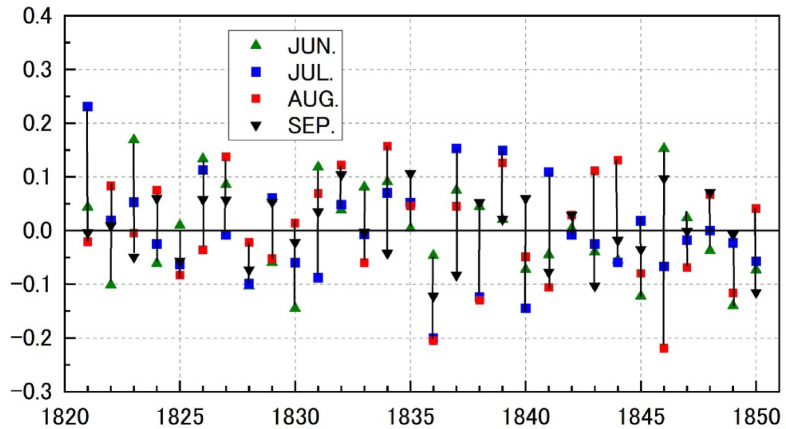
Time series of the annual scores of the PC1 of monthly solar radiation in 1821–1850, represented by the direction and length of vertical line segments. Symbols denote the scores for June (green triangle), July (blue square), August (red square), and September (black triangle pointing downward) of each year. Note that the signs for the scores for September were reversed.

The time series of the PC1 scores are shown in Fig. 6. Although the eigenvectors for the four months were not exactly the same, we combined the scores of the four months to browse the sequence. Large negative values indicated a lack of solar radiation in the zone around 32–37 °N. Remarkably, the large negative values lasted from July to September 1836 and July to August 1838, when Japan experienced years of cool summers and poor rice harvesting. The score for 1833, another well-known cool summer in the Tohoku region, was not much different from zero. It can be said that this summer had a solar radiation anomaly pattern different from that of PC1.

We do not discuss other principal components here because the spatial patterns of the eigenvectors were not coherent from one month to another, and their proportion of the variance was, at most, 17%.

### Climate variation and the market economy

Next, we examine the relationship between the price of rice in Osaka and the amount of solar radiation, as shown in Fig. 3.

Figure 3 plots the rice price trend and shows that from August 1836 to September 1837, rice prices were higher than in a typical year (50–70 monme)^24^. Contrasting this movement in rice prices with the PC1 scores reveals that the rice price trend correlates with the PC1 scores from June to September of 1836. This pattern of rice price trend correlating with PC1 scores suggests that market participants may have interpreted the lack of solar radiation between June and September 1836 (represented by the downward blue bar in Fig. 3) pessimistically and placed buy orders for rice before the October harvest in anticipation of a possible rise in rice prices.

A historical document can also verify this fact. On September 8, 1836 (the 28th day of the 7th month in the lunar-solar calendar), the Osaka Town Magistrate issued a proclamation to rice market participants stating: “Due to persistent rain and unusual climate, pessimism about the rice harvest has led to buy orders, causing rice prices to rise and impoverishing the poor. Since a poor harvest has not yet been confirmed, do not trade based on mere expectations. Instead, strive to stabilize prices at a fair level” (‘*Komeshō Kyūki*’ (old record of the rice market) from^29^). Although the term “solar radiation” is not explicitly used here, the phrase “persistent rain” in this context implies a shortage of solar radiation.

Contrary to the Shogunate’s intention to somehow stabilize the market, the rice crops were poor, and the price of rice continued to rise until the summer of 1837. One event that must be mentioned during this period was the Ōshio Heihachirō’s Rebellion. On March 25, 1837 (the 19th day of the 2nd month in the solar-lunar calendar), Ōshio Heihachirō, a bureaucrat in the Osaka Magistrate’s Office, instigated an armed uprising in Osaka City, decrying the government’s inaction toward the soaring rice prices. However, the rebellion was suppressed within a day, and it remains uncertain whether the subsequent surge in rice prices after April 4, 1837, was directly linked to it. It is plausible that the sharp rise in rice prices after the rebellion could have been influenced by market pessimism regarding a potentially poor rice harvest associated with the reduced levels of solar radiation in April.

The recovery of solar radiation from June to August 1837 coincided with a stabilization of the market trend, and rice prices began to fall in October 1837. Such a recovery of solar radiation during the rice-growing season likely bolstered the perception of a promising rice crop. Because the observed price changes occurred not only in a specific brand, such as Kumamoto rice, but consistently across all five rice brands (Fig. 3), the pattern suggests that variations in solar radiation were likely among the factors influencing rice prices during this period. Previous studies that focused on rice prices in the 12th month (around January of the next year in the modern solar calendar) did not sufficiently account for people’s perceptions of the harvest for that year’s crop.

## Discussion

### Different behavior of solar radiation and temperature

In the summer season in Japan, low values of solar radiation tend to coincide with low values of temperature, and both factors can lead to low rice yields. However, these factors do not always behave similarly.

According to alternate sources, despite moderate levels of reconstructed solar radiation in the Tohoku region during the summer of 1836, climatic conditions were anomalously cool. For example, Sekisetsu Chihō Nōson Keizai Chōsa-Sho^30^compiled a chronological table of agricultural disasters in six prefectures (administrative divisions in modern Japan) in the Tohoku region, and it recorded cool anomalies in 1836 in four of these prefectures.

The effects of solar radiation and temperature on crop yield were also different. Nishimori and Yokozawa^9^studied the climatic factors affecting rice yields in Japan using data from the modern period (1979–1994). They used multiple regression analysis to explain rice yields based on air temperature and solar radiation. Their results showed that rice yield is sensitive to temperature in the northeastern part of Japan and solar radiation in the western part.

Consequently, the climate is likely to be cooler all over Japan in years when the PC1 score of solar radiation has large positive values, even though the solar radiation anomaly in the northernmost part is small. In such years, the rice yield is likely to be lower because of low solar radiation in the western part of Japan and low temperatures in the northeastern part.

### Volcanic eruptions

Volcanic eruptions that inject a large amount of sulfur dioxide into the stratosphere will likely impact the global climate. Sigl et al.^31^list such eruptions in the time frame of our study in their database.

In the time frame of our study, the Cosigüina volcano in Nicaragua erupted in January 1835^32^, and the volcanic explosivity index (VEI) as defined by Newhall and Self^33^was 5. According to Sigl et al.^31^, the sulfur dioxide which was injected into the stratosphere is estimated to be 9 teragrams. In addition, Hutchison et al.^34^demonstrate that the Zavaritskii caldera in Simushir Island of the Kurils erupted in 1831, injecting 12 teragrams of sulfur dioxide.

Mechanisms which connect volcanic eruptions to climatic anomalies has been discussed, for example, by Robock^35^and Marshall et al.^36^. Some of the responses are straightforward. Sulfate aerosols reduce the amount of solar radiation that arrives at the surface and are likely to cause lower surface temperatures in many parts of the world. However, the responses are not limited to that. For example, the temperature anomalies in the lower troposphere in the northern middle latitudes in winter following the eruption of Pinatubo in 1991 were positive somewhere and negative elsewhere (see Plate 8 of^35^). The propagation of planetary waves in the atmosphere is likely involved. Therefore, the spatial patterns of the anomalies may be quite different according to the situation of the zonal winds, even with similar forcing.

Variations in solar radiation at the surface as reconstructed by us from historical diaries may contain some effect of volcanic forcing; however, these variations are probably not a direct effect of aerosols but an indirect effect via clouds, which planetary waves may also modulate. Also, there may be delayed responses to volcanic forcing. In such cases there must be something that works as memory, and it is likely to be heat storage in the ocean. More robust reconstructions of ocean temperature than currently available are needed in order to discuss this fully. We consider that discussing the cause-and-effect relationship between volcanic forcing and reconstructed solar radiation would be premature and that it will become fruitful when we can reconstruct global-scale patterns of climatic anomalies.

### Limitations

This study has several limitations that should be considered when interpreting the findings. First, the conversion parameters (*q*_*mean*_) derived from modern observations (1981–2010) were assumed to be applicable to the early 19th century, which may introduce some uncertainty. Second, while the number and spatial distribution of available diaries are sufficient to identify the dominant spatial mode of solar-radiation anomalies in Japan (PC1), they are insufficient to resolve higher-order modes, making it difficult to assess finer spatial variability such as that potentially relevant to the 1833 event. Third, due to data limitations, we are unable to directly observe agricultural production or distribution volumes. While historical documents suggest that climate signals played a major role in shaping price dynamics in the 1830s, rice prices may also have been affected by non-climatic factors, and we cannot entirely exclude their influence in our analysis.

## Conclusions

Despite recent advancements in climate reconstruction from tree rings and other sources that provide climate data with a resolution of approximately one month, these reconstructions are limited to early to mid-summer, posing challenges in obtaining climate or weather information for September and October, which are crucial months that impact rice yields. Moreover, while annual rice prices traditionally serve as indicators of socioeconomic conditions, disaggregating them into monthly rice prices reveals the significant impact of solar radiation from July to September on prices in the central market, exemplified by observations from 1836 to 1838, as depicted in Fig. 2.

Figure 2 shows that while solar radiation levels in July and August remained similar between 1836 and 1838, there was a notable disparity in September. In 1836, rice prices rose after September due to a lack of solar radiation. In contrast, in 1838, rice prices remained high despite the recovery of solar radiation during the same month (Fig. 3). These distinctions between 1836 and 1838 became visible with the enhanced temporal resolution of the climate and price data.

In 1836, solar radiation in July and August was approximately 10% below normal in the central part of Japan, and abnormally cool conditions prevailed across the country for three consecutive months during summer. This period coincided with a surge in rice prices starting that summer, with prices rising to three to four times the normal level (from 50-70 monme to around 200 monme) and remaining high until the summer of 1837.

Even during the Tenpō Famine, a period marked by severe food shortages, rice prices appear to have fluctuated not only from year to year but also within each year. Because climate–economy interactions are closely tied to seasonal conditions, examining climate and economic data at a monthly resolution is essential for capturing these intra-annual dynamics.

Given the nuanced relationship between climate and the economy, which is closely connected to seasonal activities, it can be useful to examine climate and economic data on a monthly basis. Moreover, establishing a nationwide rice market system makes it essential to assess climate patterns on a synoptic scale spanning thousands of kilometers; this makes it useful to examine weather description records across multiple locations to capture a broader climatic context.

Increasing spatial resolution can help historical analysis to capture interregional linkages within the national market economy, rather than treating climatic shocks as local phenomena. Enhancing temporal resolution, in turn, allows us to observe how and when people perceive or anticipate climate anomalies and adjust their behavior.

This type of historical analysis can provide context for understanding human responses to unusual climate conditions, with possible implications for contemporary societies.

## Data Availability

Historical Weather Database data are available at [http://tk2-202-10627.vs.sakura.ne.jp]; observational data from the JMA (Sect. 2.3) from 1981 to 2010 are available from the ‘Kako-no kishō data download’ (past weather data download) on the JMA web page ([https://www.data.jma.go.jp/risk/obsdl/]); Economic data are available from the “Sho Sōba no Hikae” (Record of Market Activities), which is stored in the archives of the Mitsui group in Tokyo.

## References

[1] Bolitho, H. The Tempō Crisis. In The Cambridge History of Japan, Vol. 5, the Nineteenth Century (Cambridge University Press, Cambridge, 1989).

[2] Kikuchi, I. *Ue to Shoku no Nihon-Shi* (*Japanese History of Hunger and Food*) (Yoshikawa Kōbunkan, Tokyo, [in Japanese], 2019).

[3] Brázdil, R., Pfister, C., Wanner, H., von Storch, H. V. & Luterbacher, J. Historical climatology in Europe – The state of the Art. *Clim. Change*. **70**, 363–430 (2005).

[4] Neukom, R., Steiger, N., Gómez-Navarro, J. J., Wang, J. & Werner, J. P. No evidence for globally coherent warm and cold periods over the preindustrial common era. *Nature* **571**, 550–554 (2019). 31341300 10.1038/s41586-019-1401-2

[5] Brönnimann, S. et al. Last phase of the little ice age forced by volcanic eruptions. *Nat. Geosci.* **12**, 650–656 (2019).

[6] Hirano, J. & Mikami, T. Reconstruction of winter climate variations during the 19th century in Japan. *Int. J. Climatol*. **28**, 1423–1434 (2008).

[7] Mikami, T. Climatic reconstruction in historical times based on weather records. *Geogr. Rev. Jpn Ser. B*. **61**, 14–22 (1988).

[8] Ichino, M., Mikami, T. & Masuda, K. Fluctuations of global solar radiation in Japan during the first half of the 19th century as estimated from historical weather records. *J. Geogr. (Tokyo)*. **127**, 543–552 (2018). [in Japanese with English abstract].

[9] Nishimori, M. & Yokozawa, M. Kikō Hendō Ijō Kishō Ni Yoru Nihon no Suitō Tanshū Hendō no Chiikiteki Henka (Regional change of yield per unit of paddy rice in Japan by Climatic variability and abnormal weather conditions). *Chikyū Kankyō (Glob Environ.* **6**, 149–158 (2001). https://airies.wikiplus.net/attach.php/6a6f75726e616c5f30362d326a706e/save/0/0/06_2-04.pdf [in Japanese].

[10] Brunt, L. Weather shocks and english wheat yields. *Explor. Econ. Hist.* **57**, 1690–1871. 10.1016/j.eeh.2014.12.001 (2015).

[11] Bleakley, H. & Hong, S. C. Adapting to the weather: lessons from U.S. History. *J. Econ. Hist.* **77** (3), 756–795. 10.1017/S0022050717000675 (2017). 28966394 10.1017/S0022050717000675 PMC5617122

[12] Rönnbäck, K. Climate Conflicts, and variations in prices on Pre-colonial West African markets for staple crops. *Econ. Hist. Rev.* **67** (4), 1065–1088. 10.1111/1468-0289.12058 (2014).

[13] Hamano, K. Kikō hendō no rekishi Jinkō-gaku: Tenpō no shibō Kiki o Megutte (Historical demography of climatic changes:about the mortality crisis in the Tenpō Period) in Rekishi Jinkō-gaku no Furontia (Frontiers in Historical Demography) (ed.Hayami, A., Kitō, H. & Tomobe, K.) 173–192.* Tōyō Keizai Shinpō Sha*. (2001)..

[14] Yoshimura, M. The little ice age in Japan. Historical weather data base and reconstruction of the climate in historical time. *J. Geogr. (Chigaku Zasshi)*. **102**, 131–143 (1993). [in Japanese with English abstract].

[15] Mikami, T. Long term variations of summer temperatures in Tokyo since 1721. *Geogr. Rep. Tokyo Metrop Univ.* **31**, 157–165 (1996).

[16] Iwahashi, M. *Kinsei Nihon Bukka-shi no Kenkyū* (*Studies on the History of Commodity Prices in Early Modern Japan*) (Ōhara Shinsei Sha, Tokyo, [in Japanese]. (1981).

[17] Yoshimura, M. Making the database of weather record in old diaries and its significance. *Rekishi Chirigaku (Hist Geogr.* **267**, 53–68 (2013). http://hist-geo.jp/img/archive/267_053.pdf [in Japanese with English abstract].

[18] Japan Meteorological Agency. Chijō kishō kansoku tōkei Shishin (Guideline for statistics of surface meteorological observations). (1990). https://books.google.co.jp/books?id=2g6UrgEACAAJ. [in Japanese].

[19] Ichino, M., Sakamoto, N., Masuda, K. & Mikami, T. The method for estimating global solar radiation based on weather records: toward the Climatic reconstruction in the historical period. *Tenki* **48**, 823–830 (2001). [in Japanese].

[20] Kondo, J. & Xu, J. Seasonal variations in the heat and water balances for nonvegetated surfaces. *J. Appl. Meteorol.* **36**, 1676–1695 (1997).

[21] Ichino, M., Masuda, K. & Mikami, T. Reconstruction of solar radiation for Tokyo since 1720 using weather descriptions from historical diaries. *Clim. Change*. **178**, 221 10.1007/s10584-025-04036-w (2025).

[22] Ichino, M. & Mikami, T. Spatial and Temporal differences of global solar radiation: applicability of mean daily clearness index. *Geogr. Rep. Tokyo Metrop Univ.* **38**, 15–21 (2003).

[23] Nihon Kishō Kyōkai (Japan Weather Association). *Shōwa 60 nendo NEDO itaku gyōmu seika hōkokusho* (FY1985 Commissioned Report by NEDO). New Energy and Industrial Technology Development Organization (NEDO). (in Japanese) (1986).

[24] Takatsuki, Y. *The Dojima Rice Exchange: from Rice Trading To Index Futures Trading in Edo Period Japan.* Japan Publishing Industry Foundation for Culture (2022).

[25] Bassino, J. P., Broadberry, S., Fukao, K., Gupta, B. & Takashima, M. Japan and the great divergence, 730–1874. *Explor. Econ. Hist.* **72**, 1–22 (2019).

[26] Arizono, S. *Kinsei shomin no nichijōshoku: Hyakushō wa kome o taberarenakatta ka (*The Everyday Diet of Commoners in Early Modern Japan: Were Peasants Able to Eat Rice? ). Kaiseisha. (2007).

[27] Mitsui Bunko. *Kinsei Kōki Ni Okeru Shuyō Bukka No Dōtai (Key Commodity Price Dynamics in the Late Early Modern Period of Japan)* (University of Tokyo, 1989). [in Japanese].

[28] Takatsuki, Y. & Hisamatsu, T. The role of information in the rice exchange: Yamagata Bantō’s great knowledge (1806). *Eur. J. Hist. Econ. Thought*. **30**, 395–409 (2023).

[29] Ōsaka Keizai Shiryō Shūsei Kankō Iinkai (ed.) Ōsaka Keizai Shiryō Shūsei. 4 (Ōsaka Shōkō Kaigisho, Ōsaka, [in Japanese]. (1973).

[30] Sekisetsu Chihō Nōson Keizai Chōsa-Sho. (Snowy region rural economy survey). Tōhoku chihō kyōsaku ni kansuru shiteki chōsa (Historical survey on harvest failures in the Tohoku region). Sekisetsu Chihō Nōson Keizai Chōsa-Sho, Yamagata. [in Japanese]. (1935).

[31] Sigl, M., McConnell, J. R. & Toohey, M. Volcanic stratospheric sulfur injection between 1733 and 1895 CE based on the eVolv2k-plus-D4i ice-core eruption catalogue [dataset]. *PANGAEA* (2023). 10.1594/PANGAEA.960975

[32] Longpré, M. A., Stix, J., Burkert, C., Hansteen, T. & Kutterolf, S. Sulfur budget and global climate impact of the A.D. 1835 eruption of Cosigüina volcano, Nicaragua. *Geophys. Res. Lett.* **41**, 6667–6675. 10.1002/2014GL061205 (2014).

[33] Newhall, C. G. & Self, S. The volcanic explosivity index (VEI) an estimate of explosive magnitude for historical volcanism. *J. Geophys. Res.* **87**, 1231–1238. 10.1029/JC087iC02p01231 (1982).

[34] Hutchison, W. et al. The 1831 CE mystery eruption identified as Zavaritskii Caldera, Simushir Island (Kurils). *Proc. Natl Acad. Sci. U. S. A.* **122**, e2416699122 (2025). 10.1073/pnas.2416699122 PMC11725861 39793052

[35] Robock, A. Volcanic eruptions and climate. *Rev. Geophys.* **38**, 191–219. 10.1029/1998RG000054 (2000).

[36] Marshall, L. R. et al. Volcanic effects on climate: recent advances and future avenues. *Bull. Volcanol*. **84**, 54. 10.1007/s00445-022-01559-3 (2022).

